# Antidepressant-like Effects of BDNF and NGF Individual Loop Dipeptide Mimetics Depend on the Signal Transmission Patterns Associated with Trk

**DOI:** 10.3390/ph15030284

**Published:** 2022-02-24

**Authors:** Armen G. Mezhlumyan, Anna V. Tallerova, Polina Y. Povarnina, Aleksey V. Tarasiuk, Nellya M. Sazonova, Tatiana A. Gudasheva, Sergey B. Seredenin

**Affiliations:** 1 Department of Medicinal Chemistry, V.V. Zakusov Research Institute of Pharmacology, 125315 Moscow, Russia; armezhlumyan@gmail.com (A.G.M.); antatatall@gmail.com (A.V.T.); povarnina@gmail.com (P.Y.P.); tarasiuk86@gmail.com (A.V.T.); saz-nellya@mail.ru (N.M.S.); 2 Department of Pharmacogenetics, V.V. Zakusov Research Institute of Pharmacology, 25315 Moscow, Russia; seredeninpharm@mail.ru

**Keywords:** NGF, BDNF, low-molecular weight mimetic, MAPK/ERK, PI3K/AKT, PLC, forced swim test

## Abstract

Neurotrophins are considered as an attractive target for the development of antidepressants with a novel mechanism of action. Previously, the dimeric dipeptide mimetics of individual loops of nerve growth factor, NGF (GK-6, loop 1; GK-2, loop 4) and brain-derived neurotrophic factor, BDNF (GSB-214, loop 1; GTS-201, loop 2; GSB-106, loop 4) were designed and synthesized. All the mimetics of NGF and BDNF in vitro after a 5–180 min incubation in a HT-22 cell culture were able to phosphorylate the tropomyosin-related kinase A (TrkA) or B (TrkB) receptors, respectively, but had different post-receptor signaling patterns. In the present study, we conduct comparative research of the antidepressant-like activity of these mimetics at acute and subchronic administration in the forced swim test in mice. Only the dipeptide GSB-106 that in vitro activates mitogen-activated protein kinase/extracellular signal-regulated kinase (MAPK/ERK), phosphoinositide 3-kinase/protein kinase B (PI3K/AKT) and phospholipase C-gamma (PLC_γ_) post-receptor pathways exhibited antidepressant-like activity (0.1 and 1.0 mg/kg, ip) at acute administration. At the same time, the inhibition of any one of these signaling pathways completely prevented the antidepressant-like effects of GSB-106 in the forced swim test. All the NGF mimetics were inactive after a single injection regardless of post-receptor in vitro signaling patterns. All the investigated dipeptides, except GTS-201, not activating PI3K/AKT in vitro unlike the other compounds, were active at subchronic administration. The data obtained demonstrate that the low-molecular weight BDNF mimetic GSB-106 that activates all three main post-receptor TrkB signaling pathways is the most promising for the development as an antidepressant.

## 1. Introduction

Depression is the most common mental disorder: according to WHO estimations, approximately 280 million people in the world suffer from it [1]. Modern monoaminergic antidepressants are effective in about 60% of cases and require long-term use of not less than two-three weeks to achieve a positive effect [2,3]. Therefore, the development of antidepressants with a new action mechanism is essential.

Currently, scientific studies associate the depressive disorder’s pathogenesis with impaired neuroplasticity in the hippocampus and the prefrontal cortex [4]. The central role of the brain-derived neurotrophic factor (BDNF) in neuroplasticity maintenance in these structures is well documented [5,6]. The content of BDNF in blood plasma decreases in people suffering from depression and returns to normal levels after an antidepressant treatment [7]. Postmortem studies show that the content of BDNF and its tropomyosin-related kinase B (TrkB) receptors is reduced in the prefrontal cortex and the hippocampus in suicide victims [8,9]. A decrease in the nerve growth factor (NGF), as well as BDNF level, was found in the blood plasma of people suffering from depression [10] and also in the hippocampus of suicide victims [11].

Animal studies show both BDNF and NGF to be active in various experimental models of depression, wherein BDNF is active even with a single administration [12], while a chronic administration is required for the manifestation of the NGF antidepressant-like effect [13].

It was found that the BDNF administration into the rat midbrain increases the level of serotonin and its metabolite, 5-hydroxyindoleacetic acid [14], and enhances serotonergic transmission, contributing to the growth and survival of serotonergic axons [15]. It is noteworthy that this effect was not observed for NGF. Additionally, it has been shown that the activation of neurogenesis processes by antidepressants depends on BDNF and TrkB. Thus, in mice, knocked out for the BDNF and TrkB receptor genes, proliferation, neurogenesis, and neuronal survival are not enhanced by antidepressant administration [16,17,18]. Therefore, neurotrophins, especially BDNF, are considered attractive targets for the development of antidepressants with a new mechanism of action. However, full-sized neurotrophins as proteins have a low ability to penetrate the blood–brain barrier and are unstable in biological fluids. Therefore, their use in clinical practice is limited, and the attention of researchers is attracted by the low-molecular mimetics of neurotrophins [19,20]. BDNF mimetic 7,8-dihydroxyflavone was found to have antidepressant activity [21,22].

Based on the original hypothesis—that the different loops of neurotrophins are responsible for the different physiological functions and the side chains of amino acid residues of the dipeptide central fragment of the beta-turns are “pharmacophore elements” (since as the most exposed outside, they occupy a geometrically beneficial position for interaction with the receptor [23,24])—several dimeric dipeptide mimetics of the individual NGF and BDNF loops were designed and synthesized at the V.V. Zakusov Research Institute of Pharmacology (RF Patent NO. 2410392, 2011; US Patent NO. 9683014 B2, 2017; CN Patent NO. 102365294 B, 2016; EP 2397488, 2019; India Patent 296506, 2018).

**Figure 1:**
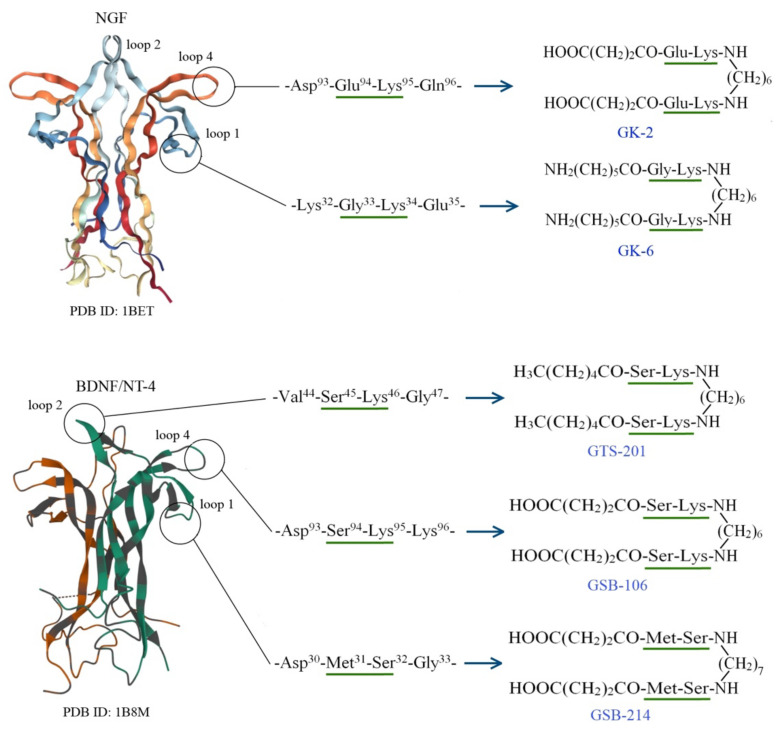
Design and modeling of the brain-derived neurotrophic factor (BDNF) and nerve growth factor (NGF) dipeptide mimetics.

Mature neurotrophins are symmetrical homodimers (Figure 1). Each protomer consists of 118–119 amino acid residues. It contains seven β-strands linked by three exposed β-turn loops (loops 1, 2, and 4, residues 28–36, 43–49, and 91–98, respectively) and an exposed loop 3 consisting of three successive reverse turns (residues 59–75) [25,26,27]. The β-turns sequences of the NGF loops 1 and 4 (-Lys32-Gly33-Lys34-Glu35) and (-Asp93-Glu94-Lys95-Gln96-), respectively, and the BDNF loops 1, 2, and 4 (Asp30-Met31-Ser32-Gly33-), (-Val44-Ser45-Lys46-Gly47-), and (-Asp93-Ser94-Lys95-Lys96), respectively, were selected as a modeling basis (Figure 1). It is interesting to note that the participation of some amino acid residues of BDNF β-turns central dipeptide fragments in interaction with the Trk receptor is consistent with the data on site-directed mutagenesis, specifically Lys^95^ (fourth loop) and Ser^45^-Lys^46^ (second loop) [28]. In the mimetics design process, β-turn central dipeptide fragments (marked bold) were retained, the preceding amino acid residue was replaced with its bioisoster, and the next one was represented with the amide group (Figure 1). Two β-turn mimetics of the corresponding loop were combined with a hexa- or heptamethylenediamine spacer, as neurotrophins are known to interact with the Trk receptors in the dimeric form.

The following dimeric dipeptide compounds were received: GK-6 (bis-[N-aminocaproyl-glycyl-l-lysine] hexamethylenediamide) and GK-2 (bis-[N-monosuccinyl-l-glutamyl-l-lysine] hexamethylenediamide), as the mimetics of the NGF loops 1 and 4, respectively [23]; and compounds GSB-214 (bis-[N-monosuccinyl-l-methionyl-l-serine] heptamethylenediamide), and GTS-201 (bis-[N-hexanoyl-l-seryl-l-lysine] hexamethylenediamide) [29], GSB-106 (bis-[N-monosuccinyl-l-seryl-l-lysine] hexamethylenediamide) [24], as the mimetics of the BDNF loops 1, 2 and 4, respectively.

In vitro studies by Western blot analysis have revealed that the NGF mimetics selectively activated the TrkA receptor, while the BDNF mimetics activated the TrkB receptor [23,29,30]. At the same time, the mimetics of different loops have different post-receptor signaling pathway activation profiles when incubated with hippocampal HT-22 cells from 5 to 180 min in vitro (Table 1). For the NGF mimetics: GK-2 (loop 4) activates phosphoinositide 3-kinase/protein kinase B (PI3K/AKT) and phospholipase C-gamma (PLC_γ_) pathways [23,31]; GK-6 (loop 1) activates PI3K/AKT, mitogen-activated protein kinase/extracellular signal-regulated kinase (MAPK/ERK), and PLC_γ_ pathways [23,31]. Regarding the BDNF mimetics: GSB-214 (loop 1) activates PI3K/AKT and PLC_γ_ [30,31]; GTS-201 (loop 2)—MAPK/ERK and PLC_γ_ [29,31]; and GSB-106 (loop 4) activates all three main signaling cascades [30,31].

The antidepressant-like activity was previously shown for one of the BDNF dipeptide mimetics, compound GSB-106 [24], and this effect depended on the TrkB activation [32]. The antidepressant-like effects of GSB-106 were shown not only in behavioral despair tests, but also in social defeat stress and chronic unpredictable stress models in rodents [32,33].

This study aims to comparatively investigate the antidepressant-like activity of the NGF and BDNF dipeptide mimetics in the Porsolt forced swim test at acute and subchronic administration and to analyze the dependence of the mimetic antidepressant effects from the pattern of its post-receptor signaling pathways activation, registered after 5–180 min incubation in an HT-22 cell culture.

## 2. Results

### 2.1. GSB-106 Was the Only Mimetic That Possesses Antidepressant-like Activity at Acute Administration

First, we investigated a possible antidepressant-like activity of the dipeptide NGF and BDNF mimetics upon their acute administration. To achieve this goal, we used the forced swimming test in BALB/c mice conducted separately for each mimetic.

One-way analysis of variance (ANOVA) of the immobility time revealed the significant treatment effect in the experiment with GSB-106 (F(3,29) = 6.416, *p* = 0.0018). Subsequent intergroup comparison with Dunnett’s post hoc test showed that GSB-106 at the doses of 0.1, 1.0 mg/kg intraperitoneally (ip.) significantly reduced immobility time (*p* = 0.0050, *p* = 0.0109, respectively) by 23.3% and 19.8% (Table 2). These results are consistent with the data previously obtained for the GSB-106 antidepressant-like activity in the forced swim test at acute administration [24]. Amitriptyline at the dose of 10 mg/kg ip. significantly reduced the immobility time (*p* = 0.0009, *p* = 0.0026) by 27.9% and 34.7% in the experiments with GSB-106 and GK-6, respectively (Table 2). The other NGF and BDNF mimetics studied did not affect the immobility time (Table 2).

So, the dipeptide GSB-106 was the only mimetic that possesses an antidepressant-like activity at acute administration.

### 2.2. At Subchronic Administration, Not Only GSB-106 but Also GSB-214, GK-2 and GK-6 Exhibit Antidepressant-like Activity

Next, we investigated a possible antidepressant-like activity of the NGF and BDNF dipeptide mimetics at their 5 day administration in the same test, but carried out at a single point of time for all compounds, except GSB-106.

GSB-106 significantly reduced the immobility time by 15.8% compared to the control group (*p* < 0.05; Student’s *t*-test) in the forced swim test at subchronic administration at the dose of 1.0 mg/kg (Table 3).

One-way ANOVA of the immobility time revealed the significant effect of treatment in the experiments with the other neurotrophin mimetics (F(7,56) = 3.934, *p* = 0.0015). Subsequent intergroup comparison with Dunnett’s post hoc test showed that among the BDNF mimetic GSB-214 had the antidepressant-like effect at subchronic administration at the dose of 1.0 mg/kg, reducing the immobility time (*p* = 0.0021) by 18.2%, and the dipeptide GTS-201 was inactive (Table 3).

The NGF mimetics GK-2 at the dose of 1.0 mg/kg and GK-6 at the dose of 2.0 mg/kg were also active and reduced the immobility time (*p* = 0.0414, *p* = 0.0184, respectively) by 13.2% and 14.7%, respectively. Amitriptyline at the dose of 10 mg/kg reduced the immobility time (*p* = 0.0122) by 15.4% (Table 3).

### 2.3. The PI3K Inhibitor Prevents the Antidepressant-like Effect of GSB-106

In the previous in vitro studies, we have shown that GSB-106 activates all the three major TrkB-associated signaling pathways—PI3K/AKT, MAPK/ERK, and PLC_γ_ (Table 1) [34]. To assess the dependence of the GSB-106 antidepressant-like effects on the post-receptor TrkB signaling pathway, we conducted a pharmacological inhibitory assay in the mice forced swimming test. Previously, we have found [32] the Trk receptor blocker K252A and the PLC inhibitor U73122 completely prevent the antidepressant-like effects of GSB-106, as well as amitriptyline.

In the present study, we examined whether LY294002, the PI3K inhibitor, affects the antidepressant-like effects of GSB-106 and amitriptyline.

**Figure 2:**
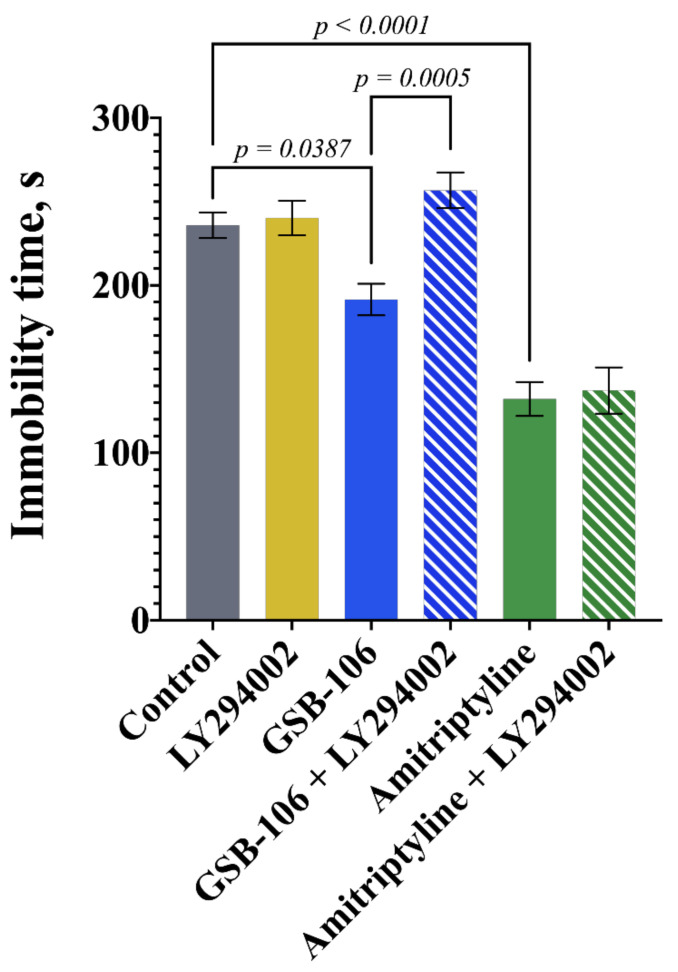
The phosphoinositide 3-kinase (PI3K) inhibitor LY294002 eliminates the antidepressant-like effect of the BDNF mimetic GSB-106 in the forced swimming test and does not affect the effect of amitriptyline. The results are presented as means ± SEM. Statistical analysis: two-way ANOVA (Tukey’s post hoc test); *n* = 10 mice per group.

As shown in Figure 2, LY294002 significantly blocked the effects of GSB-106, but not amitriptyline, on mice’s immobility time. The two-way ANOVA revealed the significant treatment effects by GSB-106 or Amitriptyline (F(2,53) = 57.72, *p* < 0.0001), PI3K inhibition by LY294002 (F(1,53) = 8.644, *p* = 0.0049) and these factors interaction (F(2,53) = 5.766, *p* < 0.0054). Tukey’s post hoc test exposed statistically significant intergroup differences. Compared with the control group, the dipeptide GSB-106 (0.1 mg/kg, ip) and amitriptyline (10.0 mg/kg, ip) significantly reduced the mice immobility time (*p* = 0.0387, *p* < 0.0001, respectively) by 18.8% and 44.0%. LY294002 at the selected dose did not decrease the mice immobility time (Figure 2). The administration of LY294002 completely prevented the antidepressant-like effect of GSB-106: there were statistically significant differences between the “GSB-106 + LY294002” group and the “GSB-106” group (*p* = 0.0005) and no differences with the control group. The PI3K inhibitor LY294002 did not affect amitriptyline activity (Figure 2).

### 2.4. The MEK1/2 Inhibitor Prevents the Antidepressant-like Effect of GSB-106

In the next test, we examined whether the mitogen-activated protein kinase kinases 1/2 (MEK1/2) inhibitor PD98059 affects the activity of GSB-106 and amitriptyline in the mice forced swimming test.

**Figure 3:**
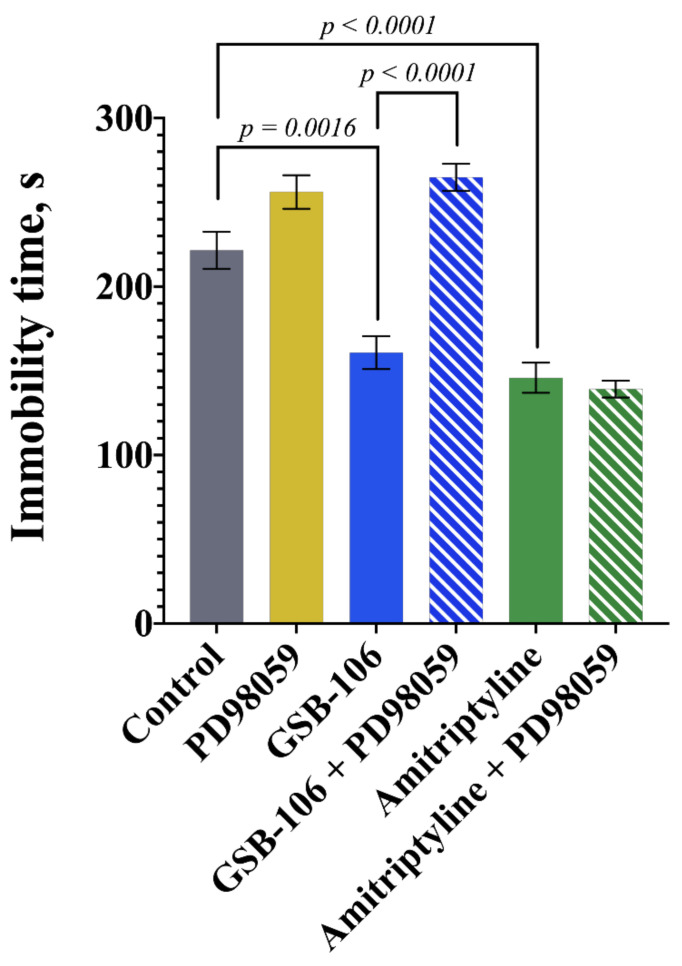
The mitogen-activated protein kinase kinases 1/2 inhibitor PD98059 eliminates the antidepressant-like effect of the BDNF mimetic GSB-106 in the forced swimming test and does not affect the effect of amitriptyline. The results are presented as means ± SEM. Statistical analysis: two-way ANOVA (Tukey’s post hoc test); *n* = 10 mice per group.

As LY294002, the PI3K inhibitor, PD98059, the mitogen-activated protein kinase kinases 1/2 (MEK1/2) inhibitor, completely abolished the antidepressant-like effect of GSB-106 (Figure 3). The two-way ANOVA of immobility time revealed the significant effects of treatment (F(2,48) = 59.95, *p* < 0.0001), MEK1/2 inhibition (F(1,48) = 34.93, *p* < 0.0001) and treatment × MEK1/2 inhibition interaction (F(2,48) = 21.71, *p* < 0.0001). GSB-106 at the dose of 0.1 mg/kg and amitriptyline at the dose of 10.0 mg/kg had the antidepressant-like effect of reducing the mice immobility time (*p* = 0.0016, *p* < 0.0001, respectively) by 27.4% and 34.2%; PD98059 did not decrease the mice immobility time (Figure 3). The administration of PD98059 completely prevented the antidepressant-like effect of GSB-106: statistically significant differences were observed in the “GSB-106 + PD98059” group with the “GSB-106” group (*p* < 0.0001), and there were no differences with the control group. The administration of PD98059 had no effect on amitriptyline activity (Figure 3).

## 3. Discussion

The forced swim test is one of the most used screening methods to identify compounds with an antidepressant-like activity. Despite the differences in the action mechanisms, various groups of antidepressants used in clinical practice (tricyclic antidepressants, selective monoamine reuptake inhibitors, etc.) are active in this test [35,36].

In the present study, it was revealed that, among of all studied NGF and BDNF dipeptide mimetics, only the BDNF loop 4 mimetic GSB-106 exhibited antidepressant-like activity at acute administration. Unlike the other BDNF mimetics, GSB-106 in vitro activates all three major TrkB-associated signaling pathways, PI3K/AKT, MAPK/ERK, and PLC_γ_ (Table 1), as well as the full-length neurotrophin [34]. The obtained results are consistent with the literature data on the antidepressant effects of BDNF and its low-molecular weight mimetic 7,8-dihydroxyflavone after acute administration. Thus, Shirayama et al. showed that the acute intracerebral administration of BDNF caused an antidepressant-like effect in the forced swimming test in rats [12]. The TrkB receptor agonist 7,8 dihydroxyflavone, which activates PI3K/AKT, MAPK/ERK, and PLC_γ_ [37,38,39] also exerted antidepressant-like activity at acute administration in the chronic social stress experiment in mice [22]. The necessity of PI3K/AKT, MAPK/ERK, and PLC_γ_ activation for antidepressant-like effects of the GSB-106 at acute administration was confirmed by a pharmacological inhibitory analysis. Previously, we found [32] the Trk receptor blocker K252A and the PLC inhibitor U73122 completely prevent the antidepressant-like effect of GSB-106 in the forced swim test. In the present study, the antidepressant-like effect of the GSB-106 was shown to be completely prevented by the inhibition of the PI3K/AKT or MAPK/ERK cascades too. It is interesting to note that the antidepressant-like effect of BDNF upon acute administration, as that of GSB-106, is abolished by the inhibition of MAPK/ERK signaling [12]. At the same time, conventional antidepressants do not affect the phosphorylation of the TrkB Tyr515, the docking site of the shc adaptor protein [40,41,42], which mediates the activation of PI3K/AKT and MAPK/ERK post receptor [43]. In accordance with the literature data, the present study did not reveal the effect of PI3K and MEK1/2 inhibitors on the antidepressant effects of amitriptyline.

One of the PI3K/AKT cascade components is the mammalian target of rapamycin (mTOR), a regulator of ribosome biogenesis and protein translation, playing an important role in synaptogenesis and synaptic plasticity [44,45]. The activation of the AKT/mTOR cascade enhances the synaptic proteins synthesis followed by an increase in the number and function of synapses that contributes to the rapid elimination of depression symptoms [46]. The MAPK/ERK signaling cascade is also involved in the rapid antidepressant-like effects mediated by mTOR [47]. The MAPK-interacting serine/threonine-protein kinase 1 and 2 (MNK1/2) are established to activate the mTOR target—eukaryotic translation initiation factor 4E (eIF4E) —due to its dissociation from the cytoplasmic FMR1 interacting protein 1 (CYFIP1), which acts as a translational repressor [48]. The depressive-like behavior is observed in mice with mutant eIF4E at the MNK1/2 phosphorylation site [47]. Thus, our results correspond to the literature data on the involvement of the PI3K/AKT and MAPK/ERK pathways of TrkB receptor signal transduction in the antidepressant-like activity implementation at acute BDNF mimetics administration.

As for the NGF dipeptide mimetics, none of the studied compounds had an antidepressant-like effect in the forced swimming test at acute administration, even the loop 1 mimetic, GK-6, which in vitro activated all three major post-receptor signaling pathways. This difference between NGF and BDNF mimetics is probably related to the differences in the TrkA and TrkB expression in the brain structures involved in the depression pathogenesis. It is known [49] that the TrkB receptors are predominantly expressed in the hippocampus and the cerebral cortex, while the TrkA receptors are practically absent there.

GSB-106 and GSB-214 exhibited antidepressant-like properties in the experiments with the subchronic administration of BDNF mimetics. The activity of GSB-214, activating in vitro only the PI3K/AKT and PLC_γ_ pathways can be explained by the vital role of the PI3K/AKT/mTOR pathway in neuroprotection, synaptogenesis, and synaptic plasticity, as well as in counteracting cell apoptosis, as described above. Moreover, the effects of BDNF mimetics at subchronic administration can be driven by the hippocampal neurogenesis contribution through the neuroblasts survival increase upon PI3K/AKT activation [50,51]. It is remarkable that GSB-106, as BDNF, promotes the survival of serum-deprived neuronal-like cells by counteracting cell apoptosis through the activation of the TrkB-dependent, mostly PI3K/AKT-associated, pro survival mechanisms, including the inactivation of the pro-apoptotic BAD protein and the suppression of caspases-9 and 3/7 [52]. However, it seems that the activation of the PI3K/AKT pathway is not enough to produce an antidepressant-like effect after a single administration of BDNF dipeptide mimetics. The BDNF mimetic GTS-201 in vitro activating MAPK/ERK and PLC_γ_ was inactive, what can be explained by the inability of the mimetic to activate PI3/AKT-associated neuroprotective and anti-apoptotic mechanisms. Notably, GTS-201 lacks neuroprotective activity in vivo [53].

The NGF mimetics GK-6 and GK-2 that in vitro activate PI3K/AKT, MAPK/ERK, PLC_γ_ and PI3K/AKT, PLC_γ_, respectively, exhibited antidepressant-like activity when administered subchronically. It can be explained by their ability to stimulate BDNF synthesis in like the full-length neurotrophin. Exogenous NGF is known to increase the expression of BDNF in the brain [54]. The ability to increase BDNF expression was also shown for the NGF dipeptide mimetic GK-2 in in vitro experiments (Antipova T.A., unpublished data). According to the literature, antidepressant-like effects of NGF are realized at subchronic, but not at acute administration. Thus, a 14 day subcutaneous NGF injection in Flinders Sensitive rats (a genetic model of depression in animals) is known [13] to promote a statistically significant active swimming time increase in the forced swim test. However, a single intracerebral injection of NGF, unlike BDNF, did not have an antidepressant-like effect in the forced swim test [12].

Thus, it was found that the most promising compounds for development as antidepressants are the low molecular weight BDNF mimetics activating all the main post-receptor TrkB signaling pathways, PI3K/AKT, MAPK/ERK, and PLC_γ_.

## 4. Materials and Methods

### 4.1. Animals

The experiments were carried out using 376 male BALB/c mice weighing 18–20 g, obtained from the Animal Breeding Facility Branch Stolbovaya (Moscow region). The animals were kept in a vivarium with natural day/night cycle and free access to standard pelleted food and water. Substance administration and behavioral tests were performed between 2:00 pm and 6:00 pm.

The study was conducted complied with the requirements of GOST 33215-2014 “Guidelines for accommodation and care of animals. Environment, housing and management” (http://protect.gost.ru/document.aspx?control=7&id=202494; accessed on 10 January 2022) and Directive 2010/63/EU of the European Parliament and of the Council of 22 September 2010 “On the Protection of Animals Used for Scientific Purposes”. All the experiments were approved by the Institutional Animal Care and Use Committee of V.V. Zakusov Research Institute of Pharmacology, Moscow (order number 3 of 18 February 2021).

### 4.2. Chemicals

#### 4.2.1. Tested Compounds

The dimeric dipeptide mimetics of neurotrophins were synthesized at the Department of Medicinal Chemistry of V.V. Zakusov Research Institute of Pharmacology as described previously [20,23,24]. **GK-2**: MW = 830.92, purity = 97.4%, [α]^22^_D_ = −47.0° (c 0.1; H2O), m.p.120–128 °C (dec.); **GK-6**: MW = 712.97, purity = 96.0%, [α]^22^_D_ = −23.3° (c 1; H2O); **GSB-106**: MW = 746.85, purity = 99.2%, [α]^21^_D_ = −42.3° (c 1; H2O), m.p.153–161 °C; **GSB-214**: MW = 766.92, purity = 95.5%, [α]^25^_D_ = +9.0° (c 0.4; DMF), m.p.162–163 °C; **GTS-201**: MW = 742.99, purity = 98.0%, [α]^25^_D_ = −14.9° (c 0.6; MeOH), m.p.110–125 °C (dec.).

The neurotrophin mimetics were studied at the doses of 1.0 mg/kg for GSB-214 and GTS-201; 0.5, 1.0, and 5.0 mg/kg for GK-2; and 2.0 mg/kg for GK-6. The doses of the NGF and BDNF mimetics were selected based on the previous studies of pharmacological activity [23,30,55]. All mimetics were dissolved in normal saline and injected intraperitoneally (ip). The volume of the injection was 5 mL/kg of body weight.

#### 4.2.2. Drug Reference Standard and Placebo

A classic tricyclic antidepressant amitriptyline was used as the reference drug at the dose of 10.0 mg/kg ip [56,57] (solution for iv. and im. administration produced by the Federal State Unitary Enterprise “Moscow Endocrine Plant”, Russia, Moscow; series 20518).

A 0.9% sodium chloride solution (normal saline) produced by OOO Mosfarm, Russia, Moscow, series 0130119, was injected ip (in volume of 5 mL/kg of body weight) to the animals of the intact control group as a placebo.

#### 4.2.3. Signaling Pathway Blockers

The PI3K inhibitor (LY294002) and the MEK1/2 inhibitor (PD98059) were obtained from the Sigma company (U.S.A.). The inhibitors were dissolved in 5% (0.25 g/kg of body weight) dimethyl sulfoxide (DMSO) solution and injected ip at the following doses: LY294002—5.0 mg/kg [58] and PD98059—2.5 mg/kg [59].

### 4.3. The Forced Swim Test

The test setup consisted of five transparent plastic cylinders with a diameter of 10 cm and a height of 30 cm, separated by black plastic opaque partitions to prevent the visual contact of animals during the study. A black plastic panel was installed behind the cylinders as a background. The cylinders were filled with 22 °C water by two-thirds so that the animals could not lean on the cylinder bottom with their paws or tail. The test was carried out either in the original technique according to Porsolt [60] with one 6 min session, or in a modified configuration [61] with two sessions performed with 24 h interval: a 10 min pretest and a 5 min test. The experiment was recorded on a video camera; the resulting video materials were processed using the ANY-maze program (Stoelting Co., Dublin, Ireland) with the calculation of the animals’ total immobility time.

#### 4.3.1. Design of the Experiment with the Acute Administration of the Mimetics

The modified technique of the forced swimming test was used. After 1 h after the pretest, the animals were ip injected with the studied mimetic, or amitriptyline, or normal saline. After 24 h after the administration, the mice were repeatedly placed in the vessel with water and their behavior was recorded for 5 min followed by the total immobility time calculation.

#### 4.3.2. Design of the Experiments with the Subchronic Administration of the Mimetics

As in the case of the acute administration, the modified technique of the forced swim test with the pretest was used. Mimetics, amitriptyline, or normal saline were administered ip daily at the same time for five days. The 10 min pretest was performed 24 h after the last injection and the 5 min test was conducted after 24 h.

### 4.4. Pharmacological Inhibitory Analysis

The forced swim test without pretest with the single injection of GSB-106 and the inhibitor was used to study the mechanism of the GSB-106 antidepressant-like effect. The inhibitors of MEK1/2 (PD98059) and PI3K (LY294002) were administered 30 min before GSB-106. The test was carried out 1 h after the GSB-106 administration. The time intervals were chosen following the literature data [62,63,64,65].

### 4.5. Statistical Analysis

Statistical processing of the results was carried out with the Prism program (GraphPad Software Inc, U.S.A., San Diego, California). The data on the antidepressant-like activity of the compounds were tested for normal distribution by the Shapiro—Wilk test. Comparisons between groups were performed using Student’s *t*-test when two groups compared, or the one-way analysis of variance (ANOVA), followed by post hoc Dunnett’s test when three or more groups compared. Pharmacological inhibitory analysis data were statistically assessed by two-way ANOVA with multiple pairwise comparisons using Tukey’s multiple comparisons test. The differences were considered statistically significant at *p* < 0.05.

## 5. Conclusions

Among all studied NGF and BDNF dimeric dipeptide mimetics, only the BDNF mimetic GSB-106, activating three major post-receptor signaling pathways PI3K/AKT, MAPK/ERK, and PLC_γ_, was shown to exhibit antidepressant-like activity at acute administration. That may be associated with synaptogenesis stimulation through mTOR. Moreover, the lack of the NGF mimetics activity is most likely associated with a low density of TrkA receptors in the hippocampus and cortex, the main brain regions involved in the pathogenesis of depression. At subchronic administration, the BDNF mimetic GSB-214 activating PI3K/AKT and PLC_γ_ was also active, which presumably may be associated with the increase in the contribution of neurogenesis through neuroblast survival augmentation due to the neuroprotective effect mediated by PI3K/AKT. At subchronic administration, the dipeptide mimetics of NGF were active in contrast to an acute administration, which can be explained by the BDNF synthesis stimulation.

## Figures and Tables

**Table 1:** Post-receptor signaling pathways activation patterns by NGF and BDNF dipeptide mimetic in in vitro studies.

Neurotrophins Mimetics	Dipeptide Code	Basis Hairpin Loop	Activation of Trk Receptor and Post-Receptor Signaling Pathways
NGF mimetics	GK-6	1st	TrkA, PI3K/AKT, MAPK/ERK, PLC_γ_ [23,31]
	GK-2	4th	TrkA, PI3K/AKT, PLC_γ_ [23,31]
BDNF mimetics	GSB-214	1st	TrkB, PI3K/AKT, PLC_γ_ [30,31]
	GTS-201	2nd	TrkB, MAPK/ERK, PLC_γ_ [29,31]
	GSB-106	4th	TrkB, PI3K/AKT, MAPK/ERK, PLC_γ_ [30,31]

**Table 2:** NGF and BDNF dipeptide mimetics activity in the forced swim test in BALB/c mice at acute administration.

Group	Dose, mg/kg, i.p.	Immobility Time, s ^1^	Immobility Time, % of Control
Control	0	220.8 ± 13.8	100
GSB-106	0.1	169.4 ± 8.2 *	76.7
GSB-106	1.0	177.0 ± 11.2 *	80.2
Amitriptyline	10.0	159.2 ± 9.8 *	72.1
Control	0	226.1 ± 6.7	100
GSB-214	0.1	223.8 ± 8.5	98.9
GSB-214	1.0	230.9 ± 7.5	102.1
Control	0	228.5 ± 9.7	100
GTS-201	0.1	241.3 ± 11.3	105.6
GTS-201	1.0	209.8 ± 10.0	91.8
GTS-201	5.0	228.1 ± 9.3	99.8
Control	0	214.4 ± 15.3	100
GK-2	0.5	234.8 ± 9.5	105.1
GK-2	1.0	225.3 ± 4.7	111.3
Control	0	204.1 ± 15.9	100
GK-6	1.0	216.8 ± 13.1	106.2
GK-6	2.0	176.9 ± 18.0	86.7
GK-6	5.0	202.6 ± 9.5	99.3
Amitriptyline	10.0	133.2 ± 10.4 *	65.3

^1^ Data are expressed as means ± standard error of mean (SEM). * *p* < 0.05 compared to the control group (one-way ANOVA (Dunnett’s post hoc test)). *n* = 7–10 mice per group.

**Table 3:** NGF and BDNF dipeptide mimetics activity in the forced swim test in BALB/c mice at subchronic administration (5 days).

Group	Dose, mg/kg, i.p.	Immobility Time, s ^1^	Immobility Time, % of Control
Control	0	205.0 ± 9.1	100
GSB-106	1,0	172.7 ± 11.4 ^#^	84.2
Control	0	242.6 ± 6.6	100
Amitriptyline	10,0	205.2 ± 8.4 *	84.6
GSB-214	1,0	198.4 ± 8.1 *	81.8
GSB-201	1,0	218.0 ± 7.7	89.9
GK-2	0,5	225.5 ± 10.5	93.0
GK-2	1,0	210.5 ± 4.4 *	86.8
GK-2	5,0	239.3 ± 6.8	98.6
GK-6	2,0	207.0 ± 11.0 *	85.3

^1^ Data are expressed as means ± SEM. ^#^ *p* < 0.05 compared to the control group (Student’s *t*-test). * *p* < 0.05 compared to the control group (one-way ANOVA (Dunnett’s post hoc test)). *n* = 8 mice per group.

## Data Availability

Data is contained within the article.
